# Missed Intensive Nursing Care Scale: Results From an Italian Validation Study

**DOI:** 10.1111/nicc.70044

**Published:** 2025-04-29

**Authors:** Ilaria de Barbieri, Martina Dato, Lisa Grego, Xiuni Gan, Elisa Daniele, Claudia Casumaro, Mayra Veronese, Matteo Danielis

**Affiliations:** ^1^ Laboratory of Studies and Evidence Based Nursing, Department of Cardiac, Thoracic, Vascular Sciences and Public Health University of Padua Padua Italy; ^2^ Chief Nurse Office, Department of the Health Care Professions Azienda Ospedale Università Padova Padua Italy; ^3^ Nursing Department The Second Affiliated Hospital of Chongqing Medical University Chongqing China; ^4^ Cardiology Unit Azienda Ospedale Università Padova Padua Italy

**Keywords:** cross‐cultural validation, intensive care units, measurement scale, missed nursing care, unfinished nursing care

## Abstract

**Background:**

Unfinished Nursing Care (UNC) refers to essential patient care that is postponed or neglected, significantly impacting outcomes such as increased morbidity, mortality and hospital‐acquired infections. In Intensive Care Units (ICUs), the complexity of patient conditions results in higher UNC rates, particularly for basic care interventions. The Missed Intensive Nursing Care Scale (MINCS) assesses the frequency and types of missed care in these settings.

**Aim:**

This study aimed to translate, culturally adapt and validate MINCS for use in the Italian ICU context, ensuring its psychometric robustness.

**Study Design:**

A methodological research for translation, cross‐cultural adaptation and validation was conducted in two hospitals in north‐eastern Italy, involving general, neurosurgical and cardiothoracic ICUs. The process included translation, back‐translation, expert evaluation, pilot testing and psychometric analysis of MINCS‐Italy (MINCS‐IT) using Cronbach's alpha, Exploratory Factor Analysis (EFA) and Rasch analysis.

**Results:**

A total of 135 ICU nurses participated in the study, 76.3% were female, and an average ICU experience of 11.1 years. The final version of MINCS‐IT contained 48 items, divided into three sections: demographics, elements of missed nursing care (34 items, *α* = 0.92), and reasons for missed care (14 items, *α* = 0.94). EFA revealed a five‐factor structure for elements of missed care (53.2% variance explained) and a two‐factor structure for reasons (64.9% variance explained). Rasch analysis supported item validity, except for one item (“Assessing patient nutritional status”), which showed suboptimal values.

**Conclusions:**

The MINCS‐IT is a reliable tool for assessing missed nursing care in Italian ICUs, addressing both fundamental and complex patient needs. Its comprehensive approach supports targeted interventions to improve care quality.

**Relevance to Clinical Practise:**

The MINCS‐IT enables nurse managers to identify missed care patterns, fostering improvements in nursing practises and patient‐family care outcomes, ultimately elevating ICU standards.

**Summary**

- What is known about the topic
  ○ Unfinished nursing care is a prevalent issue across all healthcare settings, with significant implications for patient outcomes.
  ○ Existing tools for assessing missed care primarily focus on physiological needs, often neglecting psychological and social dimensions critical to comprehensive nursing care.
- What this paper adds
  ○ The MINCS‐IT is a robust tool that encompasses dimensions beyond basic care, including emotional and esteem needs.
  ○ The MINCS‐IT offers valuable insights into missed care patterns and causes in Italian ICUs, supporting targeted interventions to enhance care quality and patient–family outcomes.

## 1 Introduction

Unfinished Nursing Care (UNC) broadly refers to situations where nurses postpone or neglect essential aspects of patient care [1]. The term UNC encompasses three conceptual traditions: tasks left undone [2], implicit rationing of care [3], and missed care [4]. Evidence from the literature highlights that UNC can significantly affect patient outcomes, including increased morbidity and mortality rates, adverse events, and hospital‐acquired infections in general hospital wards [5, 6, 7, 8]. This issue is particularly pronounced in intensive care units (ICUs), where patients' complex conditions demand more nursing attention compared to surgical or medical units [9, 10]. In ICUs, basic care interventions are the most frequently rationed, with a missed care rate of 50.1% [9]. UNC in ICUs leads to higher burnout and turnover amongst nurses, compromises communication, and may increase healthcare costs, reducing care quality [11]. Key factors contributing to UNC in ICUs include high patient acuity, increased nurse workload and inadequate staffing levels [9]. As patient acuity rises, the likelihood of UNC increases, as nurses often struggle to meet all demands amid overwhelming workloads [12, 13].

## 2 Background

To date, several tools have been developed to evaluate UNC [14, 15, 16, 17]. The Task Undone Scale (TU) was the first tool created to assess unfinished care, specifically focusing on tasks that nurses were unable to complete during their last shift due to time constraints [15]. A well‐known tool is the MISSCARE Survey, developed by Kalisch et al. [14], which evaluates the frequency of missed nursing care by identifying any nursing activities that are either partially or entirely omitted, or significantly delayed. The MISSCARE survey has been validated across different countries and settings, demonstrating its effectiveness in capturing the nuances of missed care [18, 19, 20, 21]. Another important tool is the Basel Extent of Rationing of Nursing Care (BERNCA) scale, developed and validated in Switzerland through a multicentre study by Schubert et al. [16] This scale was designed to measure the implicit rationing of nursing care, specifically the withholding or failure to perform essential nursing interventions for patients.

Recently, Yang et al. [22] developed and validated the Missed Intensive Nursing Care Scale (MINCS) as a comprehensive tool to assess the frequency and types of missed nursing care in ICUs. The scale is divided into three sections: Part A gathers demographic and professional data; Part B focuses on missed care, categorised into five dimensions based on Maslow's hierarchy of needs—physiological, safety, emotional, esteem and cognitive needs; Part C identifies reasons for missed care, across four categories: labour resources, material resources, communication and managerial factors. Unlike previous tools, which primarily focus on physiological needs, MINCS also considers psychological and social dimensions, offering a more comprehensive view of missed care. Its psychometric evaluation shows excellent validity and reliability, making it a valuable tool for addressing missed care in ICU settings. Given its comprehensive nature and demonstrated reliability, we have decided to test this tool in the Italian intensive care context to address nursing care quality. Nurse managers can use the results to design interventions aimed at enhancing the overall quality of care and ultimately improving patient and family satisfaction.

### 2.1 Aims of the Study

The aims of this study were to translate, to culturally adapt, and to validate the MINCS for use in the Italian context, evaluating its validity and internal consistency.

## 3 Materials and Methods

### 3.1 Design

A methodological research for translation, cross‐cultural adaptation and validation was adopted. The study is reported according to the COnsensus‐based Standards for the selection of health Measurement INstruments (COSMIN) guideline [23, 24].

### 3.2 Setting

This study examined two hospitals in north‐eastern Italy: a hub with three specialised ICUs (general [24 beds], neurosurgical [10 beds] and cardiothoracic [10 beds]) totalling 44 beds, and a spoke hospital with a single 20‐bed general ICU. This setup reflects the typical structure of Italy's hub‐and‐spoke healthcare system, where hub hospitals offer more comprehensive and specialised services compared to spoke hospitals [25]. Typically, the Italian ICU has a critical care physician to patient ratio of 1:8 and a nurse to patient ratio of 1:2 or 1:3 per shift.

### 3.3 Instrument Translation

**FIGURE 1:**
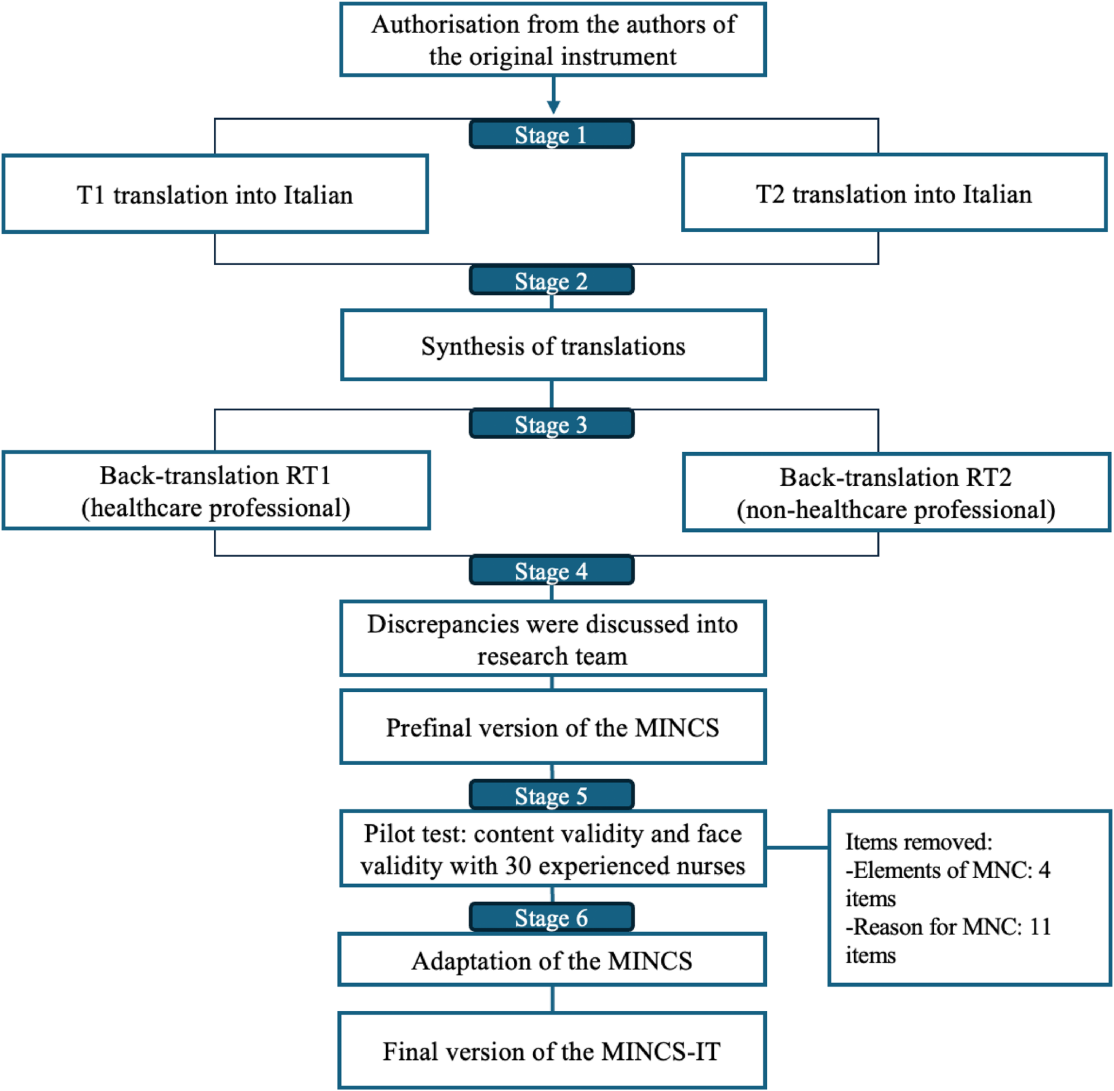
The validation procedure of the missed intensive nursing care scale—Italian version.

After getting authorisation from the author (email May 21, 2024), the instrument underwent cross‐cultural adaptation based on the methodology proposed by Beaton et al. [26] This process includes six stages: translation, synthesis, back translation, expert committee assessment, pilot testing and development of the final version (Figure 1). Initially, the MINCS was translated into Italian by two authors (IdB and LG), with discrepancies resolved through discussion with a third researcher (MV). The Italian version was then back‐translated into English to ensure it accurately reflected the content of the original [22]. Two native English‐speaking experts (one from healthcare profession and one from non‐healthcare field) independently undertook the forward and backward translation. The two English versions obtained were independently analysed and compared by two authors (IdB and LG), with any discrepancies resolved in consultation with the research team.

The research team consolidated the prefinal version, which was then pilot‐tested with 30 experienced ICU nurses. These nurses were asked to complete the MINCS to familiarise themselves with the items on the scale and assess its acceptability (i.e., whether it was appropriate to their condition and not intrusive), content validity and face validity [27, 28]. In this study, expert ICU nurses evaluated the MINCS scale to ensure its items were clear, relevant and applicable to their clinical practise. This subjective assessment by professionals in the field helps confirm the scale's apparent suitability for its intended purpose in the ICU setting [29]. Then, they assessed the MINCS' items for completeness and relevance, ensuring all essential dimensions of the measured construct were captured [29]. This process strengthens the scale's overall validity for use in ICU settings.

Following the assessment of acceptability, content and face validity, and the calculation of scores, 13 items were removed (Table S1). As a result, the instrument consists of three sections. The first section gathers demographic information such as age and sex, as well as professional details including work position, years of experience, hospital level, work department, shift type and the average number of patients cared per shift. The second section, titled ‘Elements of Missed Nursing Care in ICUs’, comprises 34 items, instead of the 38 included in the original scale, evaluated on a five‐point Likert scale (1 = never missed; 5 = always missed) as outlined by Yang et al. [22] Finally, the third section, ‘Reasons for Missed Nursing Care in ICUs’, includes 14 items, instead of the 25 included in the original scale, assessed on a four‐point Likert scale (1 = not a cause; 4 = major cause), also described by Yang et al. [22].

The final version of the Missed Intensive Nursing Care Scale—Italy (MINCS‐IT) consists of 48 items.

### 3.4 Data Collection Tools and Methods

The survey was uploaded to Microsoft Form and conducted from September 25, 2024, to October 31, 2024. Data were collected by inviting nurses to complete questionnaires during their work shifts. A facilitator (LG) used a tablet to invite nurses and assist them in filling out the questionnaires. Before completing the survey, the participants were informed about the study and were asked to sign an informed consent form. This method encouraged participation and ensured that the survey was administered in a structured and controlled environment.

### 3.5 Data Analysis

Data were analysed using IBM SPSS Statistics version 29.0 (SPSS Inc., Chicago, IL, USA) for descriptive and exploratory factor analysis and WINSTEPS software version 5.5.0.0 for Rasch analysis. For categorical variables, absolute frequencies and percentages were calculated. For continuous variables, the mean, standard deviation (SD), minimum and maximum values were reported. The psychometric properties of MINCS‐IT sections, elements and reasons were analysed separately.

The internal consistency was assessed using Cronbach's alpha and corrected item‐total correlations to evaluate the reliability of the instrument [30]. Internal consistency was considered satisfactory for alpha values > 0.70, with values ranging between 0.85 and 0.90 indicating good reliability [31].

Exploratory Factor Analysis (EFA) was performed to evaluate the underlying structure of the scale. The adequacy of the sample for factor analysis was verified using the Kaiser–Meyer–Olkin (KMO) measure and the Bartlett test of sphericity. A KMO value ≥ 0.80 was considered good. The Bartlett test was considered significant if *p* < 0.05. Principal Component Analysis (PCA) was used for factor extraction. An oblique rotation (Oblimin) was applied to allow for potential correlations between factors, consistent with the theoretical assumption of interrelated constructs [32]. The pattern matrix resulting from the oblique rotation was used to interpret the factor structure. Factor loadings of |*x*| ≥ 0.3 were deemed minimally acceptable for inclusion in a factor, whilst higher loadings (e.g., |*x*| ≥ 0.4 or |*x*| ≥ 0.5) indicated stronger and more meaningful associations.

Rasch analysis was conducted to evaluate the quality and functionality of the scale items, following the principles of item response theory [33]. The analysis included infit and outfit MeaN‐SQuare (MNSQ) statistics to assess the fit of each item to the Rasch model. Infit MNSQ is a weighted measure sensitive to responses near the person's estimated ability level, whilst Outfit MNSQ detects outlier responses to items that are exceptionally easy or difficult for the individual [34]. Optimal MNSQ values ranged from 0.5 to 1.5, with values between 1.6 and 2 considered unproductive but non‐degrading and items with MNSQ > 2 recommended for removal [35]. Additionally, the Point‐Measure Correlation Coefficient (PTMEA CORR) was calculated to evaluate whether items functioned consistently to measure the construct. Only items with positive PTMEA CORR values ranging between 0.3 and 0.8 were accepted [34].

### 3.6 Population and Sample Size

The inclusion criteria were: (i) registered nurses currently employed in an ICU; and (ii) voluntary participation in the study. Nurse students were excluded. The available target population for this study was 216 registered ICU nurses. The sample size was determined based on established guidelines for Exploratory Factor Analysis (EFA) and Rasch analysis. Although there is an ongoing debate about the ideal sample size for EFA, Kline [36] recommended sampling at least 100 subjects. Furthermore, Plichta et al. [37] suggest that EFA requires 3–10 respondents per item, or a total of 100–200 respondents. For the Rasch analysis, a sample size of at least 100 respondents is generally considered sufficient to obtain reliable and valid results. In total, 135 of 216 invited ICU nurses completed the questionnaire (response rate of 62.5%).

### 3.7 Ethical and Institutional Approvals

This study adhered to the ethical standards and principles established by the Declaration of Helsinki. Protection of personal data was guaranteed in accordance with Regulation 2016/679 of the European Parliament (GDPR) and relevant legislation on data protection. Formal authorization for the study and data collection was obtained from the Internal Review Board of two hospitals in north‐eastern Italy, on September 12, 2024, and September 23, 2024, respectively. Before participation, all individuals received detailed information about the study and provided written informed consent before completing the questionnaire. The questionnaire was anonymous to ensure participant no identifiability, and access to the data was strictly limited to the research team.

## 4 Results

The characteristics of the sample were described in Table 1. Amongst the participants, 103 were female (76.3%) with an average age of 39 years (SD = 11.2). They had an average of 11.1 years (SD = 10.7) of experience working in ICUs. The majority were employed in hub hospitals (*N* = 93; 68.9%), whilst 31.1% (*N* = 42) worked in spoke hospitals. Regarding the intensive care specialties, 71.1% (*N* = 96) worked in general ICUs, followed by 15.6% (*N* = 21) in cardiothoracic ICU, and 13.3% (*N* = 18) in neurosurgical ICU.

**TABLE 1:** Characteristics of the sample (*N* = 135).

Characteristics	
Gender, *N* (%)	
Female	103 (76.3)
Male	32 (23.7)
Age	
Mean (SD)	39 (11.2)
Median (min–max)	35 (23–66)
Working position, *N* (%)	
Staff nurse	134 (99.3)
Head nurse	1 (0.7)
Years working in ICU	
Mean (SD)	11.1 (10.7)
Median (min–max)	6 (0–40)
Hospital level, *N* (%)	
Hub	93 (68.9)
Spoke	42 (31.1)
Work department, *N* (%)	
General ICU	96 (71.1)
Neurosurgical ICU	18 (13.3)
Cardiothoracic ICU	21 (15.6)
Postgraduate course in critical care, *N* (%)	
Yes	26 (19.3)
No	106 (78.5)
Missing	3 (2.2)
Average working hours per week	
Mean (SD)	35.5 (2.78)
Median (min–max)	36 (24–45)
Shift type, *N* (%)	
On shift	119 (88.1)
Daily shift	16 (11.9)
If on shift, *N* (%)	
European shift^a^	83 (61.5)
12 h	36 (26.7)
Average number of care patients per shift	
Mean (SD)	2.6 (1.97)
Median (min–max)	2 (2–20)

Abbreviations: ICU, intensive care unit; SD, standard deviation.

^a^ Refers to a sequential shift pattern consisting of a morning shift, followed by an afternoon shift, a night shift and subsequently a rest period.

Less than one‐fifth of the sample (*N* = 26, 19.3%) were specialised in critical care nursing, whilst 78.5% (*N* = 106) were not. The median working week comprised 36 h. The majority of participants (*N* = 118, 87.4%) were shift workers. On average, nurses cared for 2.6 patients per shift (SD = 1.97), with a median of two patients.

### 4.1 Elements of Missed Nursing Care in ICUs

The overall Cronbach's alpha was high (0.92), indicating excellent reliability (Table S2). Most items demonstrated corrected item‐total correlations exceeding the acceptable threshold of 0.30, demonstrated strong correlations (Table S3). However, the item ‘Assessing patient nutritional status’ (Item 1) exhibited a low corrected item‐total correlation (0.155), suggesting limited alignment with the overall construct. Recalculation of Cronbach's alpha after hypothetical removal of this item yielded a value of 0.927, suggesting that eliminating Item 1 would have minimal impact on the scale's overall reliability. This finding indicates that no single item significantly compromised the internal consistency of the scale.

EFA reported a KMO measure of sampling adequacy of 0.871, and Bartlett's test of sphericity was significant (*χ* ^2^ = 2105.017, d*f* = 561, *p* < 0.001). The factorial structure of the Elements of missed nursing care in ICUs identified five factors associated with missed nursing care in the ICU: (1) Relationship with patients and families (11 items, 31.1% of cumulative variance), (2) Risk management and patient safety (9 items, 37.9% of cumulative variance), (3) Critical care practises (6 items, 44.1% of cumulative variance), (4) Humanising nursing care (4 items, 48.9% of cumulative variance), and (5) Patient education (4 items, 53.1% of cumulative variance) (Tables 2 and S2).

**TABLE 2:** Elements of missed nursing care in ICUs–factorial and Rasch analysis.

N. item	Elements of missed nursing care in ICU	Factorial analysis					Rasch analysis			
		1	2	3	4	5	SE	Infit MNSQ	Outfit MNSQ	PTMEA CORR
16	Communicating with family members and encouraging their involvement to harness their positive support role	**0.589**	0.19	−0.064	0.333	0.068	0.11	0.9	1.03	0.44
17	Treating patients with an attitude that is proactive, warm, patient and friendly	**0.657**	0.012	0.216	0.332	−0.002	0.10	0.94	1.00	0.55
23	Addressing patients with courtesy	**0.557**	0.067	0.512	0.108	−0.009	0.11	0.91	0.92	0.54
24	Understanding the personality traits of patients. and respecting their lifestyle habits/beliefs	**0.636**	0.194	0.293	0.184	0.135	0.11	0.89	0.87	0.54
25	Before performing nursing procedures. explaining and obtaining informed consent from conscious patients	**0.536**	0.247	0.312	−0.03	0.202	0.12	0.85	0.83	0.51
26	Protecting patient privacy during nursing procedures	**0.600**	0.282	0.305	0.143	0.167	0.14	0.74	0.84	0.46
27	Inquiring about patients' feelings after procedures and expressing gratitude and appreciation for their cooperation	**0.641**	0.253	0.136	0.036	0.216	0.12	0.76	0.81	0.48
28	Reasonably applying empathy and compassion in the workplace	**0.631**	0.114	0.253	0.215	0.042	0.11	0.76	0.79	0.56
29	Actively listening to the opinions of patients and their families	**0.597**	0.082	0.165	0.356	0.056	0.10	0.76	0.76	0.58
30	Empowering patients to fully participate in clinical diagnosis and nursing decision‐making	**0.589**	0.179	0.035	0.114	0.261	0.12	0.70	0.73	0.54
31	Assisting post‐recovery patients with functional exercises	**0.618**	0.132	−0.178	0.026	0.377	0.12	0.68	0.72	0.52
2	Assessing patient sleep quality and solve sleep‐related problem	−0.086	**0.493**	0.144	0.114	0.089	0.11	1.53	1.53	0.43
5	Turning patients according to their condition	0.191	**0.543**	0.145	0.302	−0.09	0.09	1.36	1.32	0.58
6	Strictly implementing patient identification and verification procedures	0.211	**0.365**	0.264	0.501	0.057	0.10	1.30	1.25	0.55
8	Following protocols for daily awakening of sedated patients when clinically appropriate	0.327	**0.549**	0.002	0.143	0.251	0.11	1.18	1.26	0.48
9	Maintaining accurate and comprehensive nursing documentation for each shift	0.17	**0.57**	0.118	0.053	0.067	0.10	1.24	1.25	0.48
10	Assessing the risk of adverse events in patients	0.225	**0.553**	0.236	0.139	0.087	0.29	1.20	0.80	0.31
11	Assessing indications for physical restraints in patients and implementing rational and effective restraint care	0.121	**0.687**	0.127	0.166	0.055	0.12	0.96	1.10	0.45
12	Implementing preventive nursing measures for avoiding adverse events in patients	0.162	**0.677**	0.134	0.047	0.032	0.11	0.89	1.09	0.46
21	Providing stimulatory care for comatose patients	0.225	**0.417**	−0.035	0.455	0.139	0.16	0.88	0.94	0.37
3	Ensuring patient airway patency (e.g., timely suctioning)	0.097	0.255	**0.697**	0.1	−0.078	0.22	1.11	1.45	0.29 ^*a*^
4	Providing corresponding care according to different respiratory support methods (e.g., non‐invasive/invasive ventilation)	0.129	0.256	**0.622**	−0.135	0.09	0.10	1.30	1.38	0.36
7	Carefully monitoring vital signs and changes in the patient's condition	0.206	0.344	**0.605**	−0.074	0.045	0.23	1.28	0.69	0.35
13	Executing measures for preventing and controlling hospital‐associated infections (HAIs)	0.295	0.214	**0.359**	0.102	0.281	0.10	1.01	1.07	0.48
14	Maintaining the safety of the medical equipment	0.401	0.275	**0.515**	0.177	0.186	0.09	1.07	1.06	0.58
15	Ensuring safe patient transfers	0.159	−0.02	**0.623**	0.243	0.191	0.13	1.04	0.98	0.50
18	Employing diverse and scientifically sound methods to dynamically assess the mental/psychological state of patients	0.131	0.2	−0.003	**0.686**	0.428	0.09	1.00	1.00	0.59
19	Implementing targeted and individualised care measures based on assessment results.	0.227	0.076	0.138	**0.772**	−0.024	0.12	0.97	0.99	0.47
20	Continuously evaluating the effectiveness of communication and interaction	0.256	0.356	0.019	**0.688**	−0.003	0.10	0.97	0.97	0.51
22	Creating a patient‐centered healthcare atmosphere collectively amongst department members	0.318	0.48	0.023	**0.45**	−0.019	0.11	0.94	0.94	0.53
1	Assessing patient nutritional status	−0.157	−0.17	0.435	0.169	**0.455**	0.10	2.04^a^	2.31^a^	0.23^a^
32	Encouraging patients to engage in self‐care	0.172	0.016	0.343	0.109	**0.713**	0.13	0.71	0.64	0.56
33	Daily inform patients of diagnosis. treatment/nursing plans/progress. and their medical condition	0.326	0.144	0.007	−0.006	**0.776**	0.12	0.69	0.67	0.59
34	Daily provide cognitive stimulation training by informing patients of the time, location, people, etc.	0.238	0.168	0.031	0.026	**0.708**	0.11	0.65	0.67	0.60

*Note:* (1) Relationship with patients and families; (2) Risk management and patient safety; (3) Critical care practise; (4) Humanising nursing care; (5) Patient education.

Abbreviations: ICU, intensive care unit; MNSQ, MeaN‐SQuare; PTMEA CORR, PoinT MEAsure CORRelation; SE, standard error.

^a^ Item evaluated for remotion: Infit MNSQ > 2; Output MNSQ > 2; PTMEA CORR: < 0.3.

Each factor was further analysed using Rasch analysis to examine its measurement properties. Key indicators, such as infit/outfit MeaN‐SQuare (MNSQ) statistics and point‐measure correlations (PTMEA CORR), were within acceptable ranges for most items, supporting their fit to the Rasch model (Table 2). However, the item ‘Assessing patient nutritional status’ (Item 1) showed elevated infit MNSQ (2.04), outfit MNSQ (2.31), and a low PTMEA CORR (0.23), suggesting potential misfit. Additionally, the item ‘Providing patient airway patency’ (Item 3) had a PTMEA CORR value slightly below the acceptable threshold (0.29), indicating a possible issue.

### 4.2 Reasons for Missed Nursing Care in ICUs

The overall Cronbach's alpha was high (0.94), reflecting strong internal consistency (Table S2). The corrected item‐total correlations exceeded the threshold of 0.30 for all items, ranging from 0.491 to 0.780, demonstrating their relevance to the overall construct (Table S4). Item ‘Inadequate communication and collaboration between medical and nursing staff and patients/families’ (Item 7) and item ‘Rigid nursing models’ (Item 12) showed the highest corrected item‐total correlations (0.778 and 0.780, respectively), highlighting their strong association with the total scale score. The Cronbach's alpha values remained stable when individual items were removed, ranging from 0.934 to 0.942. These findings confirm that all items make meaningful contributions to the scale, and its internal consistency is not reliant on any single item.

EFA revealed a KMO measure of sampling adequacy of 0.911, with Bartlett's test of sphericity being significant (*χ* ^2^ = 1340.021, d*f* = 91, *p* < 0.001). Factor analysis of the Reasons for missed nursing care in ICUs identified two primary factors explaining these reasons: (1) Human‐related factors (9 items, 57.3% of cumulative variance), and (2) Workplace‐related factors (14 items, 64.8% of cumulative variance) (Tables 3 and S2).

**TABLE 3:** Reasons of missed nursing care in ICU–factorial and Rasch analysis.

N item	Reasons of missed nursing care in ICU	Factorial analysis		Rasch analysis			
		1	2	SE	Infit MNSQ	Outfit MNSQ	PTMEA CORR
6	Inadequate communication and collaboration amongst nursing teams (e.g., insufficient shift handovers)	**0.688**	0.419	0.11	0.96	1.13	0.74
7	Inadequate communication and collaboration between medical and nursing staff and patients/families	**0.732**	0.413	0.11	1.07	1.11	0.76
8	Inadequate communication and collaboration amongst medical and nursing staff	**0.767**	0.359	0.11	0.89	0.97	0.78
9	Inadequate communication and collaboration with other departments/auxiliary personnel (e.g., pharmacy, logistics staff)	**0.787**	0.152	0.11	0.95	0.93	0.76
10	Frequent occurrences of nursing interruptions events	**0.684**	0.398	0.12	0.89	0.91	0.76
11	Lack of reasonable. effective. and standardised nursing processes	**0.744**	0.365	0.11	0.81	0.81	0.80
12	Rigid nursing models (e.g., failure to implement a patient‐centered nursing model)	**0.714**	0.421	0.12	0.73	0.74	0.77
13	Lack of a humanistic care philosophy in the department	**0.750**	0.219	0.12	0.59	0.57	0.78
14	Inappropriate ward layout	**0.585**	0.475	0.12	0.53	0.58	0.78
1	Occurrence of professional burnout and diminished job satisfaction amongst nursing staff	0.202	**0.605**	0.12	1.66	2.11^a^	0.57
2	Insufficient supply of departmental resources such as medications and equipment	0.242	**0.818**	0.11	1.17	1.33	0.72
3	Outdated and inconvenient‐to‐use medical equipment and devices within the department	0.407	**0.764**	0.11	1.21	1.33	0.68
4	Insufficiently intelligent electronic health record systems and health information systems	0.322	**0.775**	0.11	1.22	1.24	0.71
5	Design flaws in departmental infrastructure. such as handwashing sinks and bed unit usage area	0.425	**0.647**	0.11	1.10	1.14	0.74

*Note:* (1) Human‐related factors; (2) Workplace‐related factors.

Abbreviations: ICU, Intensive care unit; MNSQ, MeaN‐SQuare; PTMEA CORR, PoinT MEAsure CORRelation; SE, standard error.

^*a*^ Item evaluated for remotion: Infit MNSQ > 2; Output MNSQ > 2; PTMEA CORR: < 0.3.

Rasch analysis provided further support for the validity of these findings, with most items exhibiting acceptable fit statistics. However, the item ‘Occurrence of professional burnout and diminished job satisfaction amongst nursing staff’ (Item 1) demonstrated an elevated outfit MNSQ (2.11), indicating variability in responses that may warrant further investigation.

## 5 Discussion

This study aimed to translate, culturally adapt, and validate the MINCS‐IT for use in the Italian context. The process adhered to a rigorous and transparent process that has conducted to remove some items not adapted for the Italian context from the original scale.

The sample comprised mostly female nurses (76.3%) with an average of 11.1 years of ICU experience, primarily working in general ICUs of hub hospitals. 19.3% were critical care nursing specialists. The majority (87.4%) were shift workers, managing an average of 2.6 patients per shift. This experienced cohort's composition likely influenced perceptions of missed care elements and highlights the challenges ICU nurses face in providing comprehensive care. These findings are consistent with the previous study by Yang et al. [22] Both studies reported similar demographic patterns, including a high proportion of female nurses, comparable hospital levels, and relatively low specialisation rates amongst ICU nurses. The main exception was the nurse‐to‐patient ratio, with 70.9% of the sample reporting an average of three to four patients per nurse per shift [22]. A global survey spanning 34 countries revealed that 55% of ICU units implement a patient‐to‐nurse ratio of 2:1, whilst only 10% follow a 1:1 nursing care approach [38]. Similarly, a time‐point survey of 20 375 departments in 668 large general hospitals in China showed an ICU nurse‐to‐patient ratio of 1:2 [39]. These findings suggest that the nurse‐to‐patient ratio in ICUs appears to be relatively consistent worldwide, typically hovering around 1:2 or 2:1. Given this global consistency in staffing patterns, it can be inferred that a questionnaire on missed care in ICU settings could be widely applicable across different countries and healthcare systems, as staffing level is a primary antecedent of unfinished care.

The findings of this study confirmed the validity and reliability of the Italian version of the MINCS‐IT, demonstrating its applicability in the context of ICUs. With its 48 items, the MINCS‐IT showed excellent psychometric properties, with high Cronbach's alpha values for both the section assessing elements of missed nursing care (*α* = 0.92) and the section exploring reasons for missed care (*α* = 0.94). Most items had corrected item‐total correlations above the acceptable threshold of 0.30, confirming their significant contribution to the scale. The findings support the robustness of the instrument in capturing the various elements and reasons for missed nursing care in ICUs.

EFA identified a five‐factor structure for the elements of missed nursing care and a two‐factor structure for the reasons behind it. The original version of the MINCS [22] included five domains for the Elements of missed nursing care (i.e., physical, safety, emotional, esteem and cognitive) and four categories for Reasons of missed nursing care (i.e., human resources, material resources, communication and managerial factors). These factors reflect a theoretical framework of Maslow's hierarchy theory of needs, where both fundamental and more complex human needs are addressed [40, 41]. The Italian version reduced the number of items and emphasises patient education and humanization of nursing care. For years, Italian efforts have focused on improving clinical information communication to families of critically ill patients, aiming to enhance both the families' and healthcare professionals' psychological well‐being [42]. Whilst the COVID‐19 pandemic disrupted communication and support initiatives in critical care settings, the healthcare system has emerged with a renewed and intensified focus on these aspects, recognising their crucial importance and addressing them with greater determination in the post‐pandemic period.

The newly identified factors highlight the importance of addressing both basic and advanced needs. This includes nursing activities that ensure patient safety and reduce risks, such as infection prevention and timely medication administration, alongside highly technical and task‐oriented activities specific to the ICU, such as airway management and hemodynamic monitoring. Additionally, the analysis emphasizes the significance of establishing and maintaining meaningful connections with patients and their families, addressing their physical and emotional needs, as well as the provision of global and compassionate care that respects the individuality and dignity of each patient. Finally, the role of nurses in educating patients and families supports their involvement in care and enhances their health literacy.

For the reasons for missed nursing care in ICUs, emerged the encompassing challenges such as staff burnout, insufficient collaboration and communication issues amongst healthcare providers and resource availability, organisational inefficiencies and managerial barriers that hinder effective care delivery. These findings emphasise the complex interplay between human and workplace‐related factors. As highlighted in previous studies [13, 43, 44], effective managerial support, adequate resources and targeted interventions to improve team communication, resource allocation and infrastructure are associated with lower rates of missed care.

Through the Rasch analysis only one item, the ‘Assessment of the patient's nutritional status,’ showed suboptimal values, prompting consideration for its potential removal. Specifically, this item displays a low corrected item‐total correlation, high infit and outfit MNSQ values, and a low PTMEA CORR value. However, the overall robustness of the scale was not impacted, highlighting the strength of the broader construct. These findings suggest that this item may be less relevant or interpreted differently in the Italian context. One possible explanation is that Italian nurses may not perceive assessing patients' nutritional status as part of their primary competencies. This aligns with international findings, where nutritional assessment in ICUs is often overlooked [45, 46]. However, the role of nutritional assessment varies significantly depending on the clinical context. Whilst it may be considered less of a priority in acute care settings, it becomes essential in the management of chronic conditions, where nutrition plays a crucial role in maintaining and monitoring patients' overall health and preventing complications [47]. Nevertheless, existing literature highlights a recurring tension: although nurses recognise the importance of nutritional care, it is often perceived as a secondary or subordinate responsibility [48, 49, 50]. Several barriers hinder the integration of nutritional assessment into nursing practise, including limited knowledge or training in clinical nutrition, time constraints from competing priorities, and the perception of nutrition as a lower priority compared to other clinical tasks [51, 52].

## 6 Limitations

This study has some limitations. First, the use of a convenience sampling approach may have introduced selection bias, potentially affecting the representativeness of the sample and its generalizability to the broader population. Despite a high incidence rate, the sample size of 135 participants is relatively small for generalising results across Italy. With this sample, we cannot perform analyses on nurse characteristics (e.g., education levels, years of ICU experience) as predictive factors of missed care, nor investigate outcomes on care quality. Second, cultural norms and attitudes might have influenced how participants interpreted and responded to items, which could lead to differential item functioning. Lastly, whilst the total number of items was reduced from the original MINCS, the final version of the MINCS‐IT, comprising a demographic section and 48 items, may still impose a response burden on participants due to the time required to complete it.

## 7 Implications for Practice

The Italian‐validated version of the MINCS‐IT scale has the potential to measure the phenomenon of missed care from a more comprehensive perspective compared to tools developed in other contexts. This is because it incorporates dimensions of nursing care, such as those related to belongingness needs or esteem needs, which would otherwise remain invisible and unmeasurable. Historically, instruments assessing missed care have primarily focused on aspects related to basic care or those strictly connected to medical care and the diagnostic–therapeutic plan of the patient. In contrast, this new approach emphasizes dimensions more aligned with fundamental care and the core nursing domain. By using the MINCS‐IT scale, nurses and managers can maintain a heightened focus on areas specific to nursing care, fostering an approach that encompasses the patient and their family. This ensures that the unique contributions of nursing practise are effectively addressed and upheld.

## 8 Conclusions

This study successfully validated the MINCS‐IT for use in Italian ICUs. The findings demonstrated the tool's strong psychometric properties, making it a reliable and robust instrument for assessing both the elements and reasons for missed nursing care in the ICU setting. With its multidimensional approach, the MINCS‐IT provides valuable insights into the interplay between human and organisational factors that contribute to missed care. The study's results reinforce the theoretical framework of Maslow's hierarchy of needs, highlighting the importance of addressing fundamental physiological and safety needs whilst also emphasizing the need for compassionate and individualised care. By identifying factors contributing to missed care, the MINCS‐IT can guide targeted interventions aimed at optimizing nursing practises, improving work environments, and ultimately enhancing patient outcomes.

## Consent

All nurses received detailed information about the study and provided written informed consent before completing the questionnaires.

## Conflicts of Interest

The authors declare no conflicts of interest.

## Supporting information


**Data S1.** Supporting Information.

## Data Availability

The data that support the findings of this study are available from the corresponding author upon reasonable request.
