# Supplementary material for: Missed Intensive Nursing Care Scale: Results From an Italian Validation Study
Source: Nurs Crit Care. 2025 Apr 29;30(3):e70044. doi: 10.1111/nicc.70044 (PMC12038533; doi:10.1111/nicc.70044)
Supplement: Supplementary file 1 — Data S1. Supporting Information. [file NICC-30-0-s001.docx]

SUPPLEMENTARY TABLE 1 Results of face and content validity measures

| ***Item*** | | ***Face Validity*** | ***Content Validity*** |
| --- | --- | --- | --- |
| B1-1 | Assessing patient nutritional status^a^ | 2.28 | 0.70 |
| B1-2 | Evaluating the effectiveness of enteral/ parenteral nutrition*^b^* | 2.59 | 0.56 |
| B1-3 | Assessing patient thirst levels dynamically*^b^* | 2.56 | 0.60 |
| B1-4 | Implementing individualized thirst management interventions*^b^* | 2.22 | 0.63 |
| B1-5 | Assessing patient sleep quality and solve sleep-related problem | 3.97 | 0.83 |
| B2-1 | Ensuring patient airway patency (e.g., timely suctioning) | 4.58 | 1 |
| B2-2 | Providing corresponding care according to different respiratory support methods (e.g., Non-invasive/Invasive Ventilation) | 4.44 | 1 |
| B2-3 | Turning patients according to their condition | 4.63 | 1 |
| B2-4 | Strictly implementing patient identification and verification procedures | 4.07 | 0.90 |
| B2-5 | Carefully monitoring vital signs and changes in the patient's condition | 4.54 | 0.97 |
| B2-6 | Following protocols for daily awakening of sedated patients when clinically appropriate | 4.09 | 0.90 |
| B2-7 | Maintaining accurate and comprehensive nursing documentation for each shift | 4.35 | 1 |
| B2-8 | Assessing the risk of adverse events in patients (adverse events refer to falls, bed exits, unplanned extubations, etc.) | 4.42 | 1 |
| B2-9 | Assessing indications for physical restraints in patients and implementing rational and effective restraint care | 3.78 | 0.97 |
| B2-10 | Implementing preventive nursing measures for avoiding adverse events in patients | 4.32 | 1 |
| B2-11 | Executing measures for preventing and controlling hospital-associated infections (HAIs) | 4.63 | 1 |
| B2-12 | Maintaining the safety of the medical equipment | 4.07 | 0.90 |
| B2-13 | Ensuring safe patient transfers | 4.29 | 0.90 |
| B3-1 | Communicating with family members and encouraging their involvement to harness their positive support role | 3.30 | 0.90 |
| B3-2 | Treating patients with an attitude that is proactive, warm, patient, and friendly | 4.12 | 0.90 |
| B3-3 | Employing diverse and scientifically sound methods to dynamically assess the mental/psychological state of patients | 3.97 | 0.87 |
| B3-4 | Implementing targeted and individualized care measures based on assessment results, including proactive use of both verbal and non-verbal behaviors (such as touch, handshakes) for effective communication with patients^a^ | 3.06 | 0.73 |
| B3-5 | Continuously evaluating the effectiveness of communication and interaction | 3.89 | 0.93 |
| B3-6 | Providing stimulatory care for comatose patients ^a^ | 3.32 | 0.73 |
| B3-7 | Creating a patient-centered healthcare atmosphere collectively among department members | 4.57 | 0.93 |
| B4-1 | Addressing patients with courtesy | 3.96 | 0.87 |
| B4-2 | Understanding the personality traits of patients, and respecting their lifestyle habits/beliefs | 3.87 | 0.93 |
| B4-3 | Before performing nursing procedures, explaining and obtaining informed consent from conscious patients | 4.57 | 0.93 |
| B4-4 | Protecting patient privacy during nursing procedures | 4.57 | 0.97 |
| B4-5 | Inquiring about patients' feelings after procedures and expressing gratitude and appreciation for their cooperation | 3.68 | 0.93 |
| B4-6 | Reasonably applying empathy and compassion in the workplace | 3.64 | 0.87 |
| B4-7 | Actively listening to the opinions of patients and their families | 4.16 | 0.90 |
| B4-8 | Empowering patients to fully participate in clinical diagnosis and nursing decision-making | 4.04 | 0.83 |
| B4-9 | Assisting post-recovery patients with functional exercises | 3.91 | 0.97 |
| B4-10 | Encouraging patients to engage in self-care*^b^* | 3.43 | 0.57 |
| B5-1 | Explaining the necessity and safety of using various medical instruments | 3.50 | 0.83 |
| B5-2 | Daily inform patients of diagnosis, treatment/nursing plans/progress, and their medical condition | 4.10 | 0.90 |
| B5-3 | Daily provide cognitive stimulation training by informing patients of the time, location, people, etc | 3.72 | 0.90 |
| C1-1 | Inadequate competency or core capabilities among nursing staff*^b^* | 1.53 | 0.47 |
| C1-2 | Insufficient sense of responsibility and self-reliance among nursing personnel*^b^* | 1.34 | 0.50 |
| C1-3 | Occurrence of professional burnout and diminished job satisfaction among nursing staff | 3.59 | 0.93 |
| C1-4 | Limited understanding and low priority placed on patient safety and overall care accountability by nursing personnel*^b^* | 1.90 | 0.57 |
| C2-1 | Insufficient supply of departmental resources such as medications and equipment*^a^* | 3.72 | 0.77 |
| C2-2 | Outdated and inconvenient-to-use medical equipment and devices within the department | 3.65 | 0.80 |
| C2-3 | Insufficiently intelligent electronic health record systems and health information systems | 4.11 | 0.87 |
| C2-4 | Design flaws in departmental infrastructure, such as handwashing sinks and bed unit usage area | 3.99 | 0.80 |
| C3-1 | Inadequate communication and collaboration among nursing teams (e.g., insufficient shift handovers | 4.31 | 0.93 |
| C3-2 | Inadequate communication and collaboration between medical and nursing staff and patients/families | 4.48 | 1 |
| C3-3 | Inadequate communication and collaboration among medical and nursing staff | 4.51 | 1 |
| C3-4 | Inadequate communication and collaboration with other departments/auxiliary personnel (e.g., pharmacy, logistics staff) | 3.17 | 0.93 |
| C4-1 | Unreasonable allocation of nursing labor resources*^b^* | 1.94 | 0.66 |
| C4-2 | Unreasonable scheduling*^b^* | 1.76 | 0.50 |
| C4-3 | Insufficient training and assessment for nursing staff*^b^* | 1.49 | 0.40 |
| C4-4 | Insufficient emotional or financial support from management for nursing staff*^b^* | 1.01 | 0.30 |
| C4-5 | Frequent occurrences of nursing interruptions events | 4.16 | 0.93 |
| C4-6 | Unreasonable department performance management system *^b^* | 2.77 | 0.67 |
| C4-7 | Lack of supervision for inappropriate nursing behavior*^b^* | 2.38 | 0.57 |
| C4-8 | Lack of reasonable, effective, and standardized nursing processes*^b^* | 3.30 | 0.70 |
| C4-9 | Rigid nursing models (e.g., failure to implement a patient-centered nursing model) | 3.87 | 0.80 |
| C4-10 | Lack of a humanistic care philosophy in the department | 4.12 | 0.90 |
| C4-11 | Inappropriate ward layout | 2.71 | 0.87 |

*Note.* The calculation of face validity. a quantitative method was chosen through the formula of Impact Score ($Frequency(\%)\cdot Importance$). To eliminate and reduce inappropriate items. only items with an equal or higher score of 1.5 were maintained. The Content Validity calculation is also based on a quantitative of the Content Validity Index (CVI) for each item. is the results of the ratio of number of nurses who checked option 3 and 4 to total number of nurses. and the Content Validity Index for the entire scale through two methods: the Scale - Level Content Validity Index. Average ($S-\mathrm{CVI}/\mathrm{Average}=\sum(I-\mathrm{CVI})/(\mathrm{number} \mathrm{of} items)$ that calculates the average of the I-CVIs of all items and represents the average of the validity of the content for the entire scale; and the Scale – Level Content Validity Index. Universal Agreement (S-$\mathrm{CVI}/\mathrm{UA}=\sum(UA score)/(\mathrm{number} \mathrm{of} items)$ on the validity of content across the board. Respectively. in the calculation of S-CVI/UA (UA= Universal Agreement. all items that have obtained a Likert score of 3 and 4 by all respondents) a score higher than 90% is accepted.

*^a^*Item revised based on the results of the Content Validity analysis.

*^b^*Item removed based on the results of the Face and Content Validity analysis.

SUPPLEMENTARY TABLE 2 Results of Cronbach’s alfa and Exploratory Factor Analysis measures

|  | **α** | **KMO** | **Bartlett** | | | **% of cumulative variance** |
| --- | --- | --- | --- | --- | --- | --- |
|  |  |  | χ² | ***df*** | ***p-value*** |  |
| ***Elements of missed nursing care*** | 0.924 | 0.871 | 2105.017 | 561 | <.001 |  |
| *(1) Relationship with patients and families* |  |  |  |  |  | 31.11 |
| *(2) Risk management and patient safety* |  |  |  |  |  | 37.96 |
| *(3) Critical care practice* |  |  |  |  |  | 44.14 |
| *(4) Humanising nursing care* |  |  |  |  |  | 48.92 |
| *(5) Patient education.* |  |  |  |  |  | 53.16 |
| ***Reasons for missed nursing care*** | 0.940 | 0.911 | 1340.021 | 91 | <.001 |  |
| (1) Human-related factors |  |  |  |  |  | 57.35 |
| (2) Workplace-related factors |  |  |  |  |  | 64.85 |

*Note.* α= Cronbach’s alfa; *KMO= Kaiser -Meyer-Olkin; df=degree of freedom*

SUPPLEMENTARY TABLE 3 Results of corrected item-total correlations on elements of Missed Nursing Care

| ***N*** | ***Item*** | ***Corrected item-total correlations*** | ***Cronbach’s alfa if item removed*** |
| --- | --- | --- | --- |
| **1** | Assessing patient nutritional status | 0.155 | 0.927 |
| **2** | Assessing patient sleep quality and solve sleep-related problem | 0.285 | 0.924 |
| **3** | Ensuring patient airway patency (e.g., timely suctioning) | 0.397 | 0.923 |
| **4** | Providing corresponding care according to different respiratory support methods (e.g., non-invasive/invasive ventilation) | 0.357 | 0.923 |
| **5** | Turning patients according to their condition | 0.483 | 0.922 |
| **6** | Strictly implementing patient identification and verification procedures | 0.575 | 0.921 |
| **7** | Carefully monitoring vital signs and changes in the patient's condition | 0.444 | 0.923 |
| **8** | Following protocols for daily awakening of sedated patients when clinically appropriate | 0.565 | 0.921 |
| **9** | Maintaining accurate and comprehensive nursing documentation for each shift | 0.438 | 0.922 |
| **10** | Assessing the risk of adverse events in patients | 0.544 | 0.921 |
| **11** | Assessing indications for physical restraints in patients and implementing rational and effective restraint care | 0.507 | 0.921 |
| **12** | Implementing preventive nursing measures for avoiding adverse events in patients | 0.472 | 0.922 |
| **13** | Executing measures for preventing and controlling hospital-associated infections (HAIs) | 0.501 | 0.922 |
| **14** | Maintaining the safety of the medical equipment | 0.644 | 0.920 |
| **15** | Ensuring safe patient transfers | 0.420 | 0.922 |
| **16** | Communicating with family members and encouraging their involvement to harness their positive support role | 0.548 | 0.921 |
| **17** | Treating patients with an attitude that is proactive, warm, patient, and friendly | 0.573 | 0.921 |
| **18** | Employing diverse and scientifically sound methods to dynamically assess the mental/psychological state of patients | 0.558 | 0.921 |
| **19** | Implementing targeted and individualized care measures based on assessment results. | 0.479 | 0.922 |
| **20** | Continuously evaluating the effectiveness of communication and interaction | 0.575 | 0.920 |
| **21** | Providing stimulatory care for comatose patients | 0.522 | 0.922 |
| **22** | Creating a patient-centered healthcare atmosphere collectively among department members | 0.567 | 0.921 |
| **23** | Addressing patients with courtesy | 0.540 | 0.921 |
| **24** | Understanding the personality traits of patients. and respecting their lifestyle habits/beliefs | 0.660 | 0.920 |
| **25** | Before performing nursing procedures. explaining and obtaining informed consent from conscious patients | 0.563 | 0.921 |
| **26** | Protecting patient privacy during nursing procedures | 0.679 | 0.919 |
| **27** | Inquiring about patients' feelings after procedures and expressing gratitude and appreciation for their cooperation | 0.609 | 0.920 |
| **28** | Reasonably applying empathy and compassion in the workplace | 0.588 | 0.921 |
| **29** | Actively listening to the opinions of patients and their families | 0.589 | 0.920 |
| **30** | Empowering patients to fully participate in clinical diagnosis and nursing decision-making | 0.551 | 0.921 |
| **31** | Assisting post-recovery patients with functional exercises | 0.463 | 0.922 |
| **32** | Encouraging patients to engage in self-care | 0.466 | 0.922 |
| **33** | Daily inform patients of diagnosis. treatment/nursing plans/progress. and their medical condition | 0.484 | 0.922 |
| **34** | Daily provide cognitive stimulation training by informing patients of the time, location, people, etc. | 0.437 | 0.922 |

SUPPLEMENTARY TABLE 4 Results of corrected item-total correlations on reasons of Missed Nursing Care

| ***N*** | ***Item*** | ***Corrected item-total correlations*** | ***Cronbach’s alfa if item removed*** |
| --- | --- | --- | --- |
| **1** | Occurrence of professional burnout and diminished job satisfaction among nursing staff | 0.491 | 0.942 |
| **2** | Insufficient supply of departmental resources such as medications and equipment | 0.663 | 0.937 |
| **3** | Outdated and inconvenient-to-use medical equipment and devices within the department | 0.762 | 0.934 |
| **4** | Insufficiently intelligent electronic health record systems and health information systems | 0.701 | 0.936 |
| **5** | Design flaws in departmental infrastructure. such as handwashing sinks and bed unit usage area | 0.696 | 0.936 |
| **6** | Inadequate communication and collaboration among nursing teams (e.g., insufficient shift handovers) | 0.747 | 0.935 |
| **7** | Inadequate communication and collaboration between medical and nursing staff and patients/families | 0.778 | 0.935 |
| **8** | Inadequate communication and collaboration among medical and nursing staff | 0.769 | 0.935 |
| **9** | Inadequate communication and collaboration with other departments/auxiliary personnel (e.g., pharmacy, logistics staff) | 0.645 | 0.938 |
| **10** | Frequent occurrences of nursing interruptions events | 0.737 | 0.935 |
| **11** | Lack of reasonable. effective. and standardized nursing processes | 0.767 | 0.934 |
| **12** | Rigid nursing models (e.g., failure to implement a patient-centered nursing model) | 0.780 | 0.934 |
| **13** | Lack of a humanistic care philosophy in the department | 0.670 | 0.937 |
| **14** | Inappropriate ward layout | 0.714 | 0.936 |
